# Evaluation of microvessel density and p53 expression in pancreatic adenocarcinoma

**DOI:** 10.6061/clinics/2016(06)05

**Published:** 2016-06

**Authors:** Ricardo Jureidini, José Eduardo Monteiro da Cunha, Flavio Takeda, Guilherme Naccache Namur, Thiago Costa Ribeiro, Rosely Patzina, Estela RR Figueira, Ulysses Ribeiro, Telesforo Bacchella, Ivan Cecconello

**Affiliations:** I Faculdade de Medicina da Universidade de São Paulo, Departamento de Gastroenterologia; II Departamento de Patologia, São Paulo/SP, Brazil

**Keywords:** Pancreatic Neoplasms, Molecular Biology, p53 protein, Prognosis, Microvessel

## Abstract

**OBJECTIVE:**

To evaluate the prognostic significance of microvessel density and p53 expression in pancreatic cancer.

**METHODS:**

Between 2008 and 2012, 49 patients with pancreatic adenocarcinoma underwent resection with curative intention. The resected specimens were immunohistochemically stained with anti-p53 and anti-CD34 antibodies. Microvessel density was assessed by counting vessels within ten areas of each tumoral section a highpower microscope.

**RESULTS:**

The microvessel density ranged from 21.2 to 54.2 vessels/mm^2^. Positive nuclear staining for p53 was found in 20 patients (40.6%). The overall median survival rate after resection was 24.1 months and there were no differences in survival rates related to microvessel density or p53 positivity. Microvessel density was associated with tumor diameter greater than 3.0 cm and with R0 resection failure.

**CONCLUSIONS:**

Microvessel density was associated with R1 resection and with larger tumors. p53 expression was not correlated with intratumoral microvessel density in pancreatic adenocarcinoma.

## INTRODUCTION

Pancreatic ductal adenocarcinoma (PDA) is an aggressive carcinoma whose incidence and prevalence have steadily increased over the years. As a result, PDA has become the fourth most common cause of cancer-related death in the western world 1. Pancreatic cancer is also the fourth leading cause of cancer-related mortality in the United States, and in Brazil, the five-year actuarial survival rate is 5% 2. The annual death rate from the disease almost equals its annual incidence due to the aggressive nature of the cancer and the lack of effective means of screening for it during its early, curable stage. Molecular markers and imaging have not proven to be accurate modalities for screening for pancreatic cancer. The diagnosis and management of pancreatic cancer continues to be an overwhelming challenge 3,4.

Surgical treatment of PDA is considered a gold standard procedure as staging permits, but the large majority of patients have tumors that are unresectable at the time of diagnosis. Nevertheless, in the absence of credible alternative cures, surgical resection remains the only option with any potential for curing pancreatic adenocarcinoma, with overall resectability rates ranging from 15% to 20% for all patients, mortality rates ranging from 2 to 5% and total complication rates ranging from 30 to 40% 5</underline>.

Angiogenesis is the formation of new blood vessels from a pre-existing vascular network. During the prognostic state of cancer, it has been suggested that oxygen must penetrate 200 µm from a blood vessel to support cell existence and that neovascularization is mandatory for tumor cell survival 6. Some studies 7,8 have correlated microvessel density (MVD) to angiogenesis and the presence of metastasis in the peripheral regions of pancreatic tumors. The p53 tumor suppressor gene seems to regulate angiogenesis to some extent, and p53 mutations have been reported as possible markers for identifying precursor lesions with malignant transformation potential.

The aim of the current study was to determine what type of relationship exists between MVD and p53 expression with respect to patient outcome following surgical treatment of pancreatic adenocarcinoma. Furthermore, we assessed the correlation between clinicopathological features, MVD and survival outcome in these patients.

## SUBJECTS AND METHODS

This study was approved by the Research and Ethics Committee of the Faculdade de Medicina da Universidade de São Paulo. Forty-nine patients underwent duodenopancreatectomy with lymphadenectomy and histologically proven pancreatic adenocarcinomas were selected. All patients were surgically treated at the Department of Digestive Surgery, São Paulo School of Medicine and did not receive chemotherapy or anti-angiogenesis therapy before surgery. All patients were followed up and data were collected between 2008 and 2012. The cases included 22 males and 27 females, with a mean age of 58.3 years (Table 1). Paraffin blocks containing fragments of tumor and adjacent epithelia were sectioned, stained with hematoxylin & eosin (H&E), and prepared for histological analysis by light microscopy.

### Immunohistochemical determination of p53 expression

Paraffin-embedded tissues were used for immunohistochemical analysis of p53 expression. First, sections (2-3 μm) were mounted on microscope slides. Then, the sections were deparaffinized in xylene at 60^o^ C for 30 minutes, followed by treatment with a graded series of alcohol and rehydration. For the deparaffinization, the samples were incubated with hydrogen peroxide in methanol for 20 min to block endogenous peroxidase. The sections were microwaved twice at 500 W for 5 min each time in 10 mmol/L sodium citrate (pH 6.0). The sections were then incubated with antibody against human p53 protein produced from the K0680 clone (DAKO LSAB®) and washed with phosphate-buffered saline (PBS).

The slides were examined by investigators who were blinded to the corresponding clinicopathologic data. p53 immunoreactivity was assessed as positive only when tumors exhibited intense nuclear staining and the reactivity was categorized as either negative expression (less than 10% positive tumor cells) or positive expression (at least 10% positive tumor cells).

### Quantification of microvessel density

**Figure 1:**
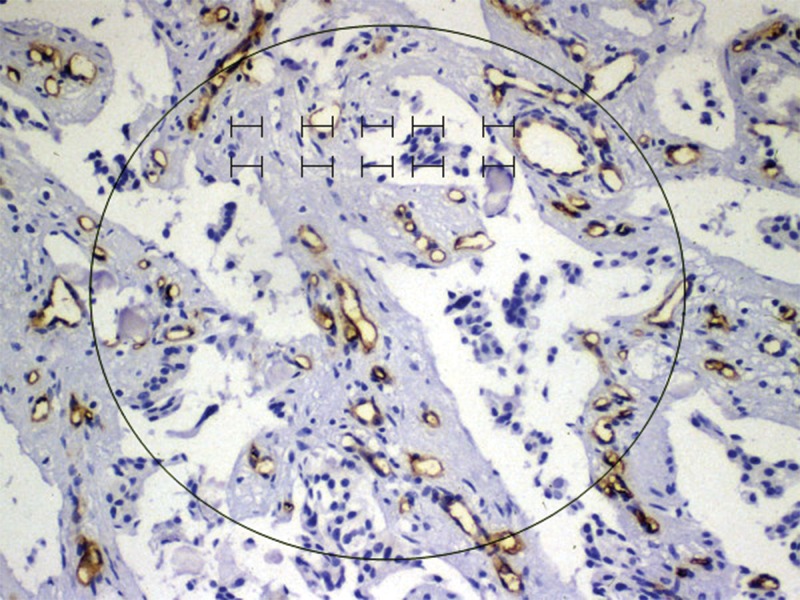
Vessel counting using reticulum identification.

To quantify microvessel density (MVD) in sections stained for CD34, entire tumor sections were scanned at low power (40x magnification) to identify areas rich in microvessels, which were then counted in ten randomized areas (Figure 1). In each area, individual microvessels were counted under high power (200x magnification) to obtain a vessel count for a hundred randomized defined points. The average vessel count in the 10 randomized areas was taken as the MVD. The vessel count was considered significant when the reticulum was in the same position as the stained endothelial cells.

### Associations with clinicopathological factors

We next studied the associations between MVD and the following clinicopathological factors: sex, age (52 years old), staging, tumor size, histological type, vascular invasion, margins and survival rates. We also assessed the relationship between p53 expression and the same clinicopathological factors. Finailly, we compared the associations identified for MVD and p53.

### Statistical methods

The univariate association between MVD and each clinicopathological item was analyzed using chi-squared tests and t-tests. To determine independent prognostic factors, a generalized linear model was used. Kaplan-Meyer and Log-Rank tests were applied to assess survival rate. *P*<0.05 was considered statistically significant.

## RESULTS

Of the 49 patients included in the study, forty-six had cephalic pancreatic carcinoma (93.8%). Thirty-one patients (63.2%) were at least stage IIB and 32 (65.3%) patients had tumors that were pT3. Twenty-nine patients (59.2%) had lymph node metastasis, 45 (91.8%) had perineural invasion and 26 (53.1%) had vascular invasion. The average tumor size was 3.36 cm (1.5 to 6.5 cm).

The mean MVD was 46.2 vessels/mm^2^, and this value was markedly higher in tumors equal to or greater than 3.0 cm in diameter (*p*=0.03) (Table 2).

Table 3 shows the relationships that were identified between p53 and clinicopathological factors; however, no correlations were found.

The generalized linear model analysis of the assessed clinicopathological factors revealed that surgical margin was the only independent factor related to DMV (*p*<0.05).

**Figure 2:**
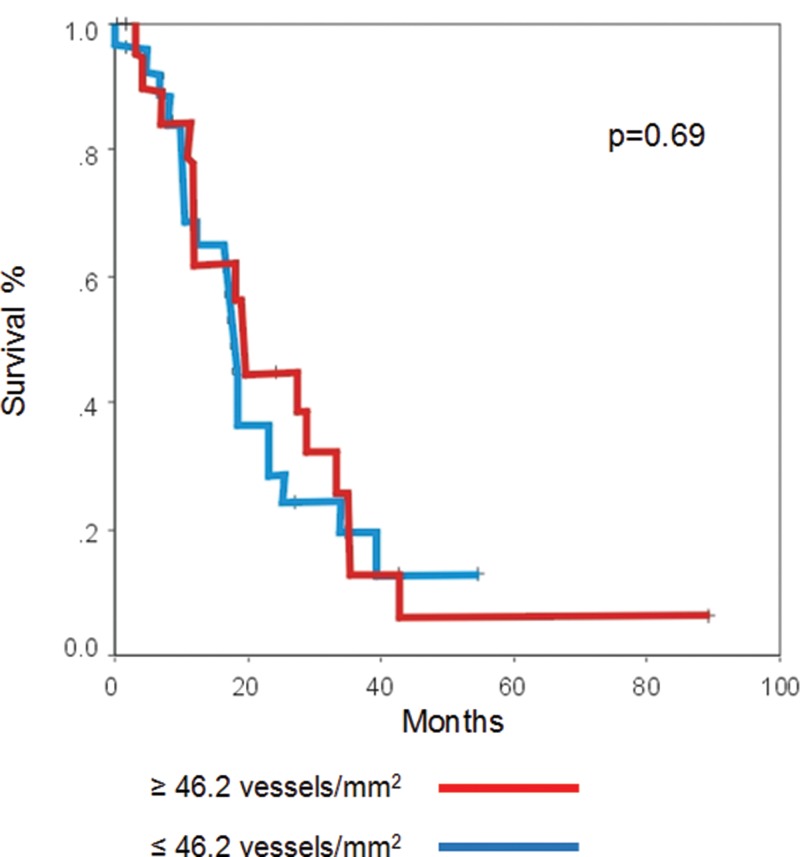
Relationship between overall survival (months) and microvessel density.

The actuarial survival rate did not demonstrate any statistically significant differences when compared to the MVD (*p*=0.69) (Figure 2).

## DISCUSSION

In 1971, Folkman formulated the hypothesis that angiogenesis in tumor specimens can be determined by the numbers and structures of blood vessels associated with a tumor and depends on neovascularization, which it is closely associated with tumor growth, staging and prognosis 9,10. In the same way, knowledge regarding the biomolecular mechanisms underlying angiogenesis has enabled efficient inhibition of this process using anti-angiogenic molecules.

Several studies demonstrated that angiogenesis precedes tumor progression and depends on a switch from a pre-vascular to a vascular phase. Recent works have shown that angiogenic factors are already expressed by pre-invasive lesions and the induction of angiogenesis is completed during the transition from hyperplasia to neoplasia. This phenomenon is important for invasive cancer screening in high-risk patients. Angiogenesis is also an important aspect of drug delivery for several tumor types 11,12.

Our study indicated that MVD is not correlated with survival prognosis in PDA, a finding that is concordant with results reported by Ellis et al. 13 and Karademir et al. 14. This result likely arose because of the absence of patient selection criteria and the different methodological methods applied (e.g., for vessel counting; Table 4), resulting in diverse MVD values.

In contrast, late diagnosis of tumors and the use of surgical treatment have resulted in advanced staging of PDA. These processes allowed a large number of microvessels to be identified during tumor analysis and this MVD could not be differentiated between groups. Kuehn et al. 15 demonstrated that a correlation exists between MVD and prognosis; however, over 60% of the patients studied were stage I and therefore had sparser MVD compared to those with advanced tumors.

The p53 tumor suppressor gene is the most frequently mutated gene in human cancer. p53 mutations play a role in determining tumor sensitivity to apoptosis-inducing treatments, such as radiation or cytotoxic drugs 16,17. p53 protein has various important functions in cellular integration, including cell growth control, response to DNA damage, cell-cycle checkpoint control, regulation of transcription and control of genomic stability. It has been suggested that p53 protein may play a role in suppressing angiogenesis 18. However, in our study, no relationship was identified between p53 and MVD or clinicopathological factors. Overall, MVD analysis can be a useful tool for patient selection when studying angiogenesis inhibition 12,19.

In conclusion, our results demonstrate that MVD is higher in tumors greater than 3.0 cm in diameter and in cases with involved margins. Furthermore, no relationship was found to exist between p53 expression and tumor angiogenesis, MVD or survival rates. Finally, neither MVD nor p53 expression could predict survival in patients with pancreatic adenocarcinoma. There was also no correlation between p53 expression and intratumoral MVD. Finally, MVD analysis is useful for predicting margin involvement.

## AUTHOR CONTRIBUTIONS

Jureidini R and Cunha JE conceived and designed the study, contributed to data acquisition, analyzed the data and wrote the manuscript. Bacchella T and Cecconello I helped designing the study and participated in manuscript revision. Takeda F, Namur GN, Ribeiro TC, Figueira ER and Ribeiro Jr.U participated in manuscript revision. Patzina R performed the pathology studies. All authors read and approved the final manuscript.

## Figures and Tables

**Table 1:** Demographic and clinicopathologic distributions.

Variable		N	%
**Gender**			
	Female	27	55.1%
	Male	22	44.9%
Age			
	Mean = 58.3 (40–77 months)		
T			
	1	5	10.2%
	2	8	16.3%
	3	32	65.3%
	4	4	8.2%
N			
	Negative	20	40.8%
	Positive	29	59.2%
Staging			
	IA	4	8.2%
	IB	5	10.2%
	IIA	9	18.4%
	IIB	27	55.1%
	III	4	8.2%
Perineural invasion			
	Absent	4	8.2%
	Present	45	91.8%
Vascular invasion			
	Absent	23	46.9%
	Present	26	53.1%
Size			
	Mean = 3.32 (1.5-6.5 cm)		
	<3.0 cm	18	36.7%
	≥3.0 cm	31	63.3%
Histological type			
	Well differentiated	23	46.9%
	Moderately differentiated	19	38.8%
	Undifferentiated	7	14.3%
Margins			
	Free	30	61.2%
	Compromised	19	38.8%
Vascular resection			
	No	39	79.6%
	Yes	10	20.4%
MVD			
	Mean		
	<46.2	28	57.1%
	≥46.2	21	42.9%
p53			
	Negative	29	59.2%
	Positive	20	40.8%

**Table 2:** Relationships between microvessel density and clinicopathological features.

		No. patients	Mean MVD	*p*
**Total**		**49**	**46.2**	
Age				
	<52 years	14 (28.6%)	47.9±16.9	0.62
	≥52 years	35 (71.4%)	45.0±21.5	
Gender				
	Male	22(44.9%)	45.1±19.2	0.71
	Female	27(45.1%)	47.1±17.2	
T				
	T1-2	13 (26.5%)	49.4±19.8	0.41
	T3-4	36 (73.5%)	44.7±17.6	
N				
	Negative	20 (40.8%)	47.3±17.6	0.18
	Positive	29 (59.2%)	45.2±18.9	
Size				
	<3.0 cm	20 (40.8%)	39.2±13.3	0.03
	≥3.0 cm	29 (59.2%)	50.4±19.8	
Histological type				
	Well and moderately differentiated	42 (85.7%)	44.7±17.2	0.05
	Undifferentiated	7 (14.3%)	55.3±12.5	
p53				
	Negative	29 (59.2%)	44.0±14.7	0.39
	Positive	20 (40.8%)	48.9±22.3	
Vascular invasion				
	Absent	23 (46.9%)	46.1±18.3	0.99
	Present	26 (53.1%)	46.2±18.4	
Margins				
	Free	30 (61.2%)	42.5±15.6	0.04
	Compromised	19 (39.8%)	53.3±20.1	

**Table 3:** Relationships between p53 expression and clinicopathological factors (no correlations were found).

		p53 negative	p53 positive	*p*
Age				
	<52 years	7	7	0.52
	≥52 years	22	13	
Gender				
	Male	12	15	0.04
	Female	17	5	
T				
	T1-2	9	4	0.21
	T3-4	20	16	
N				
	Negative	11	9	0.77
	Positive	18	11	
Size				
	<3.0 cm	11	9	0.77
	≥3.0 cm	18	11	
Histological type	Well and moderately differentiated	24	18	0.41
	Undifferentiated	5	2	
Vascular invasion				
	Absent	14	9	1.0
	Present	15	11	
Margins				
	Free	19	11	0.55
	Compromised	10	9	

**Table 4:** Different methods used to count vessels.

Author	Year	Number of patients	Antibody	Number of areas	Type of count	MVD (vessels/mm^2^)
Present study	2008	49	CD34	10	Weidner	46.2
Takagi	2008	41	CD34CD45Factor VIII	3	Lúmem	
Esposito	2004	137	CD34	5	Weidner	101*
Nakagawa	2002	32	CD34	5	Weidner	
Niedergethmann	2002	70	CD34	5	Weidner	85*
Karademir	2000	22	Factor VIII	3	Weidner	3.4#
Ikeda	1999	40	CD34	6	Weidner	
Fujimoto	1998	50	CD34	4	Weidner	30.1
Ellis	1998	22	Factor VIII	-	Weidner	44.9

* Only evaluated hypervascularized areas of neoplasm.

#Vessels/mm^2^/mm^3^ – vessels/mm.
